# A Highly Sensitive Fiber Optic Sensor Based on Two-Core Fiber for Refractive Index Measurement

**DOI:** 10.3390/s131014200

**Published:** 2013-10-22

**Authors:** José Rafael Guzmán-Sepúlveda, Rafael Guzmán-Cabrera, Miguel Torres-Cisneros, José Javier Sánchez-Mondragón, Daniel Alberto May-Arrioja

**Affiliations:** 1 Fiber and Integrated Optics Laboratory, Electronics Engineering Department, UAM Reynosa-Rodhe, Universidad Autónoma de Tamaulipas, Carr. Reynosa-San Fernando S/N, Reynosa, Tamaulipas 88779, Mexico; E-Mail: jrafael_guzmans@yahoo.com.mx; 2 NanoBioPhotonics Group, DICIS, University of Guanajuato, Salamanca, Guanajuato 368850, Mexico; E-Mails: guzmanc@ugto.mx (R.G.-C.); mtorres@ugto.mx (M.T.-C.); 3 Photonics and Optical Physics Laboratory, Optics Department, INAOE, Puebla, Puebla 72000, Mexico; E-Mail: delta_dirac@hotmail.com

**Keywords:** fiber optic sensor, refractive index sensor, two-core fiber

## Abstract

A simple and compact fiber optic sensor based on a two-core fiber is demonstrated for high-performance measurements of refractive indices (RI) of liquids. In order to demonstrate the suitability of the proposed sensor to perform high-sensitivity sensing in a variety of applications, the sensor has been used to measure the RI of binary liquid mixtures. Such measurements can accurately determine the salinity of salt water solutions, and detect the water content of adulterated alcoholic beverages. The largest sensitivity of the RI sensor that has been experimentally demonstrated is 3,119 nm per Refractive Index Units (RIU) for the RI range from 1.3160 to 1.3943. On the other hand, our results suggest that the sensitivity can be enhanced up to 3485.67 nm/RIU approximately for the same RI range.

## 1. Introduction

Refractive index (RI) sensors are suitable for applications related to quality control and industrial processing, mainly in the food industry and environmental contamination monitoring, due to their compact and simple architecture [1]. Regarding the scientific aspects, RI sensors find a wide range of applications in chemical and biological analysis, biomedical applications, and specimen detection [2]. Due to this duality of scientific and technological applications in which RI sensors have been demonstrated to achieve high-performance measurements, RI sensing stands as one of the most important techniques in the development of highly sensitive sensors.

A growing interest in fiber optic sensors for RI sensing has arisen due to their well-known characteristics such as compactness, high sensitivity, *in situ* measurements, and immunity to external electromagnetic interference. A large variety of fiber-based RI sensors can be found in the literature, the most widely studied being those based on fiber interferometry, fiber gratings, and specialty fibers. For example, RI sensors based on photonic crystal fibers (PCF) have been demonstrated to allow efficient architectures in both transmission and reflection configurations with a linear response in a relatively wide range of operation. Nevertheless, these sensors have relatively low sensitivities, on the order of 190.9 nm per Refractive Index Units (RIU) and 6.67 nm/RIU for transmission and reflection configurations, respectively [1,3]. Regarding RI sensors based on fiber gratings, several techniques to improve the sensitivity have been reported. A tilted Long Period Fiber Grating (tilted-LPFG) exhibiting highly linear response with a slope equal to 340 nm/RIU, has been reported in which the sensitivity was enhanced by improving the coupling between the core and cladding modes [4]. As for grating techniques applied to PCF, a LPFG inscribed on a PCF with a high-sensitivity of 2000 nm/RIU in RI measurements was recently demonstrated [5]. Alternative techniques such as those based on polymeric coatings have also been demonstrated to enhance the sensitivity of LPFG sensors, however these types of sensor exhibit non-linear responses, and specialized imaging equipment is required to verify the fabrication process [6,7].

Finally, regarding sensors based on fiber interferometry, some efficient alternatives have been demonstrated for RI sensing using Michelson and Mach-Zehnder interferometers. The most widely studied fabrication techniques are based on abrupt tapering and micro-machining. Although the fabrication process for abrupt tapers is simple, the overall result is a sensitivity lower than 30 nm/RIU [8–10]. On the other hand, interferometric RI sensors based on micro-machined cavities have been demonstrated to be the most sensitive for RI sensing, achieving sensitivities higher than 9,300 nm/RIU [11,12]. In similar fashion to the grating-based techniques, despite the very high sensitivity that can be achieved by this type of sensor, special equipment, such as femtosecond lasers, is required for machining the cavities. More recently, an alternative version of a RI sensor based on a twin-core fiber for RI measurements has been demonstrated using a simple setup and yielding a sensitivity of 826.8 nm/RIU [2]. However, the respectable sensitivity can only be achieved at the expense of requiring multiple bends in different sections of the fiber.

Recently, a fiber optic RI sensor based on the coupling between a partially filled hollow channel and a solid core has been proposed [13]. The sensor is demonstrated to be competitive since it has a simple architecture and it exhibits high sensitivity, 3250 nm/RIU, for RI measurement in the range from 1.5 to 1.66. The proposed sensor requires a relatively long section of fiber and large times in order to allow an interaction length sufficient to induce meaningful changes in the spectral response (10 min to fill ∼1 cm). However, the main drawbacks of the sensor are the need for a guided mode in the liquid core and the fact that the lowest measurable RI is ∼1.45, which makes this sensor not suitable for applications where aqueous solutions are to be measured.

In this work a simple, compact, and cost-effective fiber optic RI sensor based on a Two-Core Fiber (TCF) is demonstrated. The high performance characteristics of the proposed RI sensor are put to the test in the measurements of the salinity of salt water mixtures and of the water content in adulterated alcoholic beverages. The largest sensitivity experimentally demonstrated in our laboratory is 3119 nm/RIU for the RI range from 1.3160 to 1.3943.

## 2. Principle of Operation

**Figure 1.**
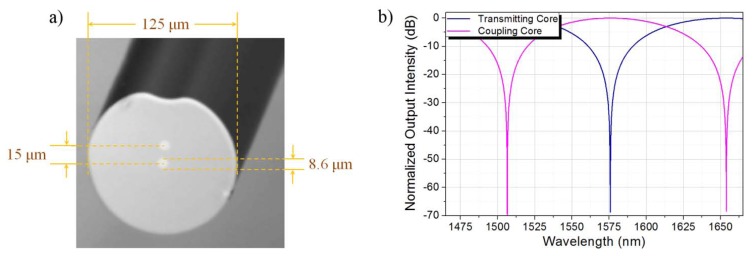
(**a**) Cross section of the TCF; (**b**) Spectral response of the TCF.

Figure 1a shows a picture of the TCF that has been used as the sensing element. The diameter of the cladding is the standard 125 μm and the two cores, both having diameter equals to 8.6 μm, are asymmetrically located: one of the cores is located at the center of the fiber while the other is located 15 μm away from the central core (center-to-center separation distance). The RI of the cladding and the cores are 1.443 and 1.448, respectively, which leads to a numerical aperture of N.A. ≈ 0.12. The TCF was manufactured at ACREO Fiberlab (Kista, Sweden), and it was designed for two main purposes: first, to allow direct splicing to standard single-mode fibers (SMF) without the need of special procedures and, second, to induce overlap between the modes of the cores thus leading the fiber to respond as a directional coupler.

**Figure 2.**
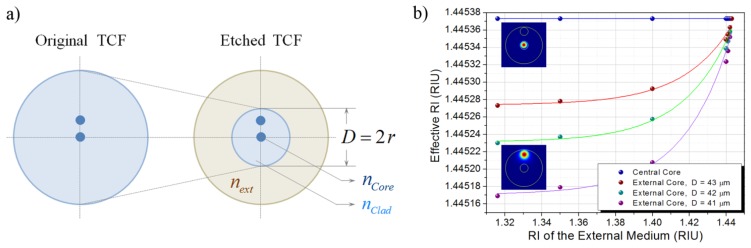
(**a**) Schematic of the TCF after the etching process; (**b**) ERI of the central and external core for TCF diameter of 41 μm, 42 μm, and 43 μm.

The analytical spectral response of the TCF in the spectral window from 1465 nm to 1665 nm is shown in Figure 1b. Since the TCF is to be spliced between two SMFs, only the central core needs to be excited at the beginning of the TCF section. Therefore, the central core is treated as the transmitting core since the TCF response will be collected by the output SMF, while the off-center core is considered the coupling waveguide. In order for the TCF to be sensitive to the surrounding media, it is proposed that the cladding of the TCF be controllably removed by means of wet chemical etching so that the TCF is capable of interacting with the external environment. Figure 2a shows a rendering of the proposed scheme. To further understand the interaction between the etched fiber and the surrounding environment, numerical simulations in COMSOL Multiphysics^®^ were performed for a structure with the following characteristics: the core diameter is kept fixed at 8.6 μm while the cladding diameter *D* of the TCF is reduced from 125 μm to 41 μm, 42 μm, and 43 μm (*i.e.*, fiber radius *r* is reduced from 62.5 μm to 20.5 μm, 21.0 μm, and 21.5 μm). The RI of the cores and the cladding of the TCF remain unaltered (*i.e.*, 1.448 and 1.443, respectively); additionally, the etched TCF is surrounded by an external medium with specific refractive index ***n****_ext_*.

Preliminary numerical simulations were focused on sketching the individual interaction between each core and the surrounding media. The effective refractive index (ERI) was calculated for both cases, when only the central core is considered and then when considering only the off-axis core, for external media with RI ranging from that of water (1.316) to that of the TCF cladding (1.443). Figure 2b shows the ERI of each core for a TCF diameter of 41 μm, 42 μm, and 43 μm respectively. It can be observed that when only the central core is considered, a constant ERI is obtained regardless of the external surrounding media, which suggests that the central core does not interact with the external environment. On the other hand, unlike the central core, the off-axis core strongly interacts with the surrounding media: the ERI directly depends on the RI of the surrounding media and it changes more rapidly as the diameter of the TCF is reduced. As expected from the structure itself, the ERI of both structures tends to the same value as the RI of the external media approaches the RI of the cladding of the TCF. Since the coupling coefficient is directly related to the optical properties of the coupled cores, any changes in the coupling between the cores are induced by variations in the ERI of the off-axis core due to the interaction with the external surrounding media.

**Figure 3.**
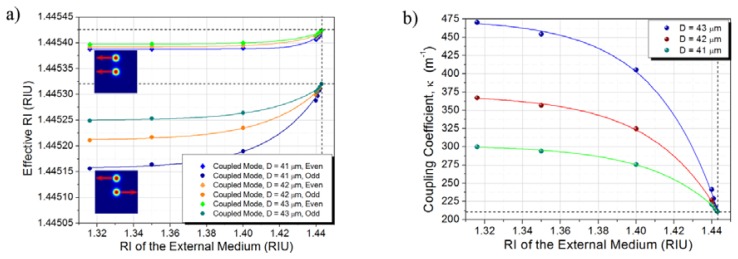
(**a**) ERI of the even and odd coupled modes; (**b**) Coupling coefficient as a function of the refractive index of the surrounding media for TCF diameter of 41, 42, and 43 μm.

The response of the TCF can then be evaluated by exploring the dependence of the coupling between the cores as a function of the RI of the surrounding media. An indirect measurement of this dependency is the variation on the ERI of the coupled modes, which can be obtained directly from the numerical simulations performed in COMSOL Multiphysics®. The ERI of the even and odd coupled modes obtained from the numerical simulations for an operation wavelength of 1550 nm for TCF diameter of 41 μm, 42 μm, and 43 μm are shown in Figure 3a. The dashed lines indicate the limit for which the refractive index of the surrounding media reaches the refractive index of the cladding of the TCF.

Once the ERI of the even and odd coupled modes are determined using numerical simulation, it is then possible to compute the corresponding coupling coefficient since it can be expressed in terms of the propagation constants of the even and odd coupled modes which, in turn, are determined by the ERI at a particular wavelength [14]:

(1) $$\kappa =\frac{\beta_{\text{even}}-\beta_{\text{odd}}}{2}=\frac{\pi }{\lambda_{0}}(n_{\text{eff},\text{even}}-n_{\text{eff},\text{odd}})$$

Figure 3b shows the coupling coefficient for the same operating wavelength (*i.e.*, 1,550 nm) as a function of the RI of the surrounding media for TCF diameter of 41, 42, and 43 μm. The coupling coefficient was calculated directly from the data shown in Figure 3a and using Equation (1). It can be noted that, for this particular conditions, the coupling coefficient between the cores decreases as the RI of the surrounding media approaches the RI of the cladding of the TCF. The dashed line indicates the limit for which the refractive index of the surrounding media approaches the refractive index of the cladding of the TCF.

Based on the results obtained from the numerical simulations, the spectral response of the TCF as a function of the RI of the surrounding media can be obtained. A very simple approach consists in evaluating Equation (2), which is the analytical expression of the electric field for a directional coupler, for all the wavelengths within the spectral window of interest. The directional coupler is assumed to have fixed length z = L, the coupling coefficient is to be recalculated for each wavelength and the difference between the propagation constants of the individual cores is taken into account through the ***δ*** parameter [15]:

(2) $$A=\cos\left(\sqrt{\kappa^{2}+\delta^{2}}z\right)+j\frac{\delta }{\sqrt{\kappa^{2}+\delta^{2}}}\sin\left(\sqrt{\kappa^{2}+\delta^{2}}z\right)$$

**Figure 4.**
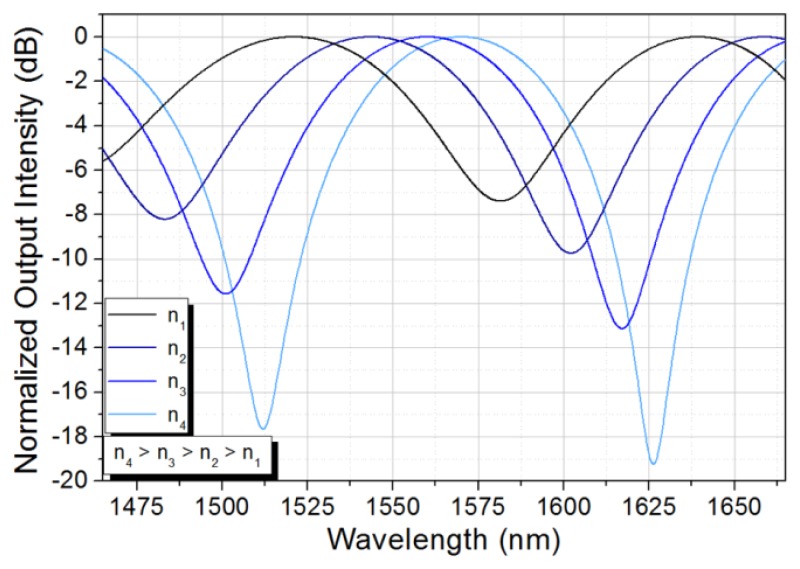
Analytical spectral response of the TCF for different surrounding media.

According to the results obtained from the numerical simulations, the spectral response of the etched TCF is expected to experience a red spectral shift as the RI of the external surrounding media is increased, as shown in Figure 4. Additionally, the contrast of the spectral response is expected to improve as the RI of the surrounding media increases. The latest is related to the fact that a more symmetric structure takes place as the RI of the external media approaches the RI of the fiber cladding. This effectively reduces the parameter ***δ*** in Equation (2) all the way to zero at the upper RI limit dictated by the TCF cladding.

Therefore, based on the results from the numerical simulations, the etched TCF structure is ideal for the realization of a RI sensor by following the spectral shifts produced by the RI of the external medium. Another advantage of the sensor is that the sensitivity can be controlled by tuning the thickness of the TCF cladding on the off-axis core.

## 3. Experimental Section

**Figure 5.**
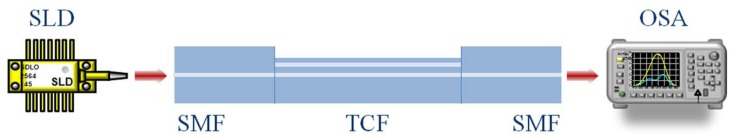
Schematic of the experimental setup for RI measurement.

A schematic of the experimental setup for RI measurement is shown in Figure 5. The setup consists of a super luminescent diode (SLD) centered at 1,580 nm, a 50 mm long section of TCF (*i.e.*, interaction length) spliced between two SMFs, and an optical spectrum analyzer (OSA). From the numerical simulations previously described it was found that only the off-axis core interacts with the external environment. Therefore, instead of removing the cladding of the entire TCF we choose to remove only the cladding around this core. From a practical point of view, removing material only from a section of the TCF will help to give the fiber good mechanical support after etching. The TCF was aligned such that the external core is facing up and then it was fixed using regular epoxy allowing the bottom of the fiber to be protected by the epoxy itself and the top of the fiber to be exposed to the etching solution. The rest of the fiber was covered with epoxy to protect it during etching and then the cladding of the TCF was slowly removed using buffered oxide etchant (BOE).

Fixing the TCF in the way just described also allows neglecting bending and surface tension effects since the fiber remains in the same position at all times thus leading to the sensor spectral response to depend only on the RI of the external environment.

### 3.1. Refractive Index of Binary Mixtures

**Figure 6.**
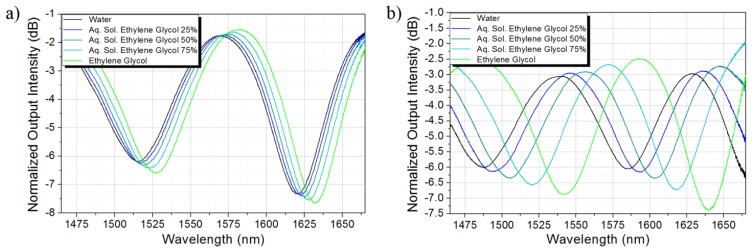
Spectral response of the RI sensor for etching times (**a**) 267 min; (**b**) 280 min.

Firstly, the sensor was tested in the RI range from 1.3160 to 1.3943, which extreme values correspond to the RI of water and ethylene glycol, respectively, at 20 °C. Intermediate values correspond to the RI of binary liquid mixtures between water and ethylene glycol with volume fractions of 0.75/0.25, 0.50/0.50, and 0.25/0.75, respectively. The RI of the mixtures was estimated by averaging several models for the quantitative determination of the RI of binary liquid mixtures [16]. Figure 6a,b shows the sensor spectral response after etching the TCF during 267 min and 280 min, respectively. As expected, the spectral response red shifts and the contrast is enhanced as the RI of the external environment increases. The wavelength shift is larger as more material from the cladding is removed around the off-axis core since a stronger interaction between the outer core and the surrounding liquid is achieved.

**Figure 7.**
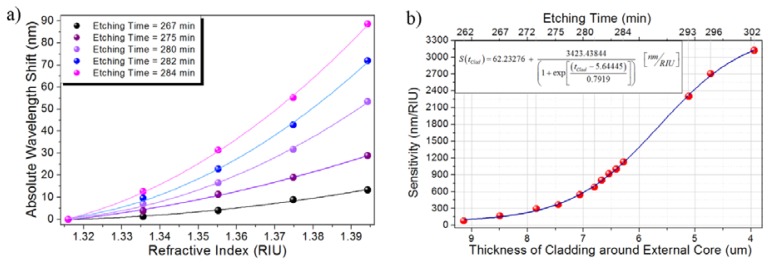
(**a**) Absolute wavelength shift of the sensor response for external media ranging from water to ethylene glycol; (**b**) Sensitivity of the RI sensor as a function of the thickness of the cladding around the off-axis core.

In order to evaluate the sensor performance after removing different amounts of material, the same set of liquids was tested for etching times ranging from 262 min to 302 min. For this range of etching times, the thickness of the cladding around the off-axis core approximately ranges from 9.14 μm to 3.94 μm, assuming an etching rate of 130 nm/min [17]. Figure 7a shows the absolute wavelength shift produced by the tested liquids for several etching times within the range from 262 min to 284 min. Despite the sensor was tested for etching times up to 302 min, the wavelength shift induced for the largest etching times was out of the spectral window where the measurement was performed so not all the liquids in the set could be measured.

The nonlinear behavior of the sensor response expected from the numerical simulations, which can be expected because the coupling coefficient does not change linearly with the external RI, was confirmed by the experimental results, as can be noticed from Figure 7a. However, in order to provide a measurement of the sensor performance, an approximate sensitivity was estimated by taking the ratio between the total wavelength shift and the total RI change [18]. Figure 7b shows the estimated sensitivity of the RI sensor as a function of the thickness of the cladding around the off-axis core, which is directly related to the etching times in the range from 262 min to 302 min.

At first, when the cladding has been thinned down enough to allow the off-axis core to interact with the external liquid, the sensor exhibits low sensitivity since a weak interaction takes place. The thinner the cladding the stronger the interaction and thus larger changes are induced to the ERI of the off-axis core. Despite the sensitivity dramatically increases, it finds its limit when the cladding has been completely removed. The numerical fitting of the sensor sensitivity as a function of the cladding thickness allows sketching the limit sensitivity to be approximately 3,485.67 nm/RIU for the RI of the external environment ranging from 1.3160 (water) to 1.3943 (ethylene glycol). The experimental results allow observing a sensitivity of 3,119 nm/RIU, which is quite close to the theoretical limit obtained from the numerical fitting of the experimental results.

### 3.2. Salinity

A different application of the developed sensor is related to the measurement of salinity in aqueous solutions [19,20]. Since it is well know that the RI of water changes with the salinity concentration, the sensor was tested for salinity measurement by preparing several sodium chloride (NaCl) aqueous solutions. The aqueous solutions were classified into two sets based on the solution concentration: high-concentration, from 0 M to 5 M, and low-concentration, from 0 M to 1 M. The RI of the solutions was calculated to vary from 1.3160 to 1.3603 and from 1.3160 to 1.3262 for the high- and low-concentration range, respectively, due to the linear dependency on the NaCl concentration at room temperature (20 °C) [11].

#### 3.2.1. High-Concentration Range

The spectral response of the sensor was experimentally measured in the concentration range from 0 M to 5 M for etching times ranging from 289 min to 302 min, which leads to the cladding around the off-axis core to have approximate thickness between 5.63 μm and 3.94 μm approximately. In similar fashion to the previous case, the spectral response red shifts as the refractive index of the external environment increases, in other words, the sensor spectral response shifts depending on the NaCl concentration of the aqueous solution and the spectral shift can be directly correlated to the solution concentration.

**Figure 8.**
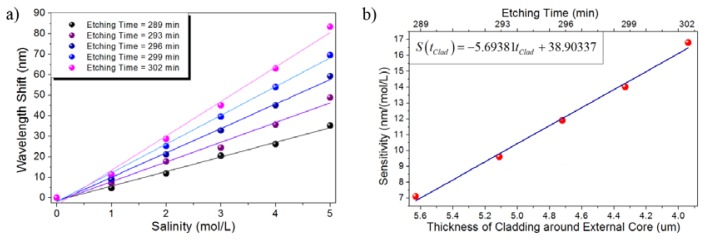
Salinity, high-concentration regime: (**a**) Absolute wavelength shift of the sensor response; (**b**) Sensitivity of the TCF sensor as a function of the remaining cladding.

Figure 8a shows the wavelength shift induced on the spectral response as a function of the NaCl concentration for several etching times. It can be clearly noticed that the larger etching times lead to stronger interactions and then longer spectral shifts are induced for the same set of solutions. In this particular case, a highly linear response is obtained for the studied cases resulting in a sensitivity ranging from 7.1071 nm/(mol/L) to 16.7914 nm/(mol/L) for etching times ranging from 289 min to 302 min. The largest wavelength shift, which was naturally obtained for the largest etching time (302 min), was measured to be 83.25 nm, as can be confirmed from Figure 8a. On the other hand, Figure 8b shows that the sensor sensitivity also shows a highly linear dependency on the thickness of the cladding around the off-axis core for the particular case of the high-concentration regime and for the tested etching times.

#### 3.2.2. Low-Concentration Range

**Figure 9.**
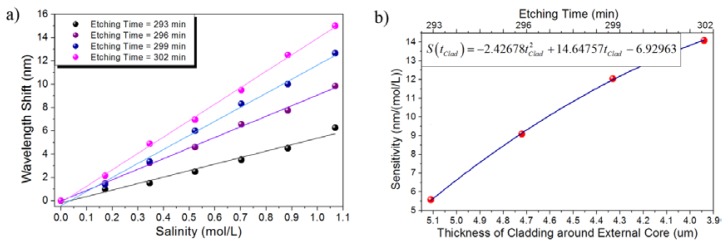
Salinity, low-concentration regime: (**a**) Absolute wavelength shift of the sensor response; (**b**) Sensitivity of the TCF sensor as a function of the remaining cladding.

After evaluating the performance of the sensor in the high-concentration range, the sensor spectral response was then tested for low-concentrated saline solutions. In this regime the concentration ranges from 0 M to 1.069 M and the sensor response was evaluated for etching times ranging from 293 min to 302 min. It can be noticed that although the spectral red shift is reduced it can still be resolved by the sensor. As shown in Figure 9a, the highly linear response is maintained and a sensitivity ranging from 5.5691 nm/(mol/L) to 14.0917 nm/(mol/L) for etching times ranging from 293 min to 302 min is achieved, respectively. Figure 9b shows that for this particular case in which low-concentration NaCl aqueous solutions were measured, the sensor sensitivity also shows a highly linear response.

Compared to the high-concentration regime, the spectral response of the sensor is expected to shift less since the low-concentration range leads to smaller RI variations. However, from the experimental results it can be noticed that the sensor response for each saline solution in the low-concentration range can be still clearly identified. The largest wavelength shift, which was obtained for the largest etching time (302 min), was measured to be 15 nm, as can be confirmed from Figure 9a.

The capability of the TCF sensor to perform salinity measurements with high sensitivity over a wide range of concentrations is demonstrated by evaluating the sensor response in both the high- and low-concentration regimes. The highest sensitivity of 16.7914 nm/(mol/L) within the measured salinity concentrations is more than 20 times larger than that recently reported for polymide-coated photonic crystal fibers and polymide-coated fiber Bragg gratings [21].

### 3.3. Adulterated Alcoholic Beverages

Adulteration of alcoholic beverages is a great concern either due to quality control of the origin or the potential harm to humans when diluted with unknown substances (mainly ethyl alcohol). Since addition of water or alcohol changes the RI of the alcoholic beverage, our sensor can be potentially used in such measurements. In this case, the sensor was tested for the authentication of alcoholic beverages by using several tequila solutions. The tequila used in the experimental measurements was Jose Cuervo Tradicional™, which we verified to be a certified tequila, and all the solutions were diluted only with water and the set of solutions ranges from pure tequila to a 50/50 volume percent diluted solution. Based on the RI of water within the spectral window of interest and the RI reported for tequila, the RI of the tested solutions is estimated to range from 1.3338 to 1.3518 [22].

The spectral response of the sensor was experimentally measured for etching times ranging from 293 min to 302 min, which leads to the cladding around the off-axis core to have approximate thickness between 5.1 μm and 3.9 μm. As expected from the previous results, both the spectral response red shifts and the contrast enhances as the refractive index of the external environment increases. In this particular case, since pure tequila is the reference liquid and diluted solutions are measured, the results can also be interpreted from the point of view that the sensor spectral response blue shifts as tequila is more diluted.

**Figure 10.**
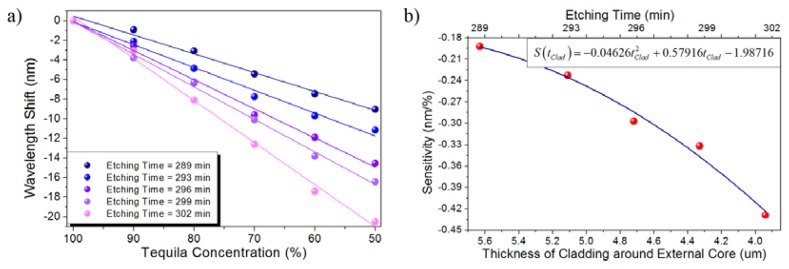
Diluted solutions of tequila: (**a**) Absolute wavelength shift of the sensor response; (**b**) Sensitivity of the TCF sensor.

Figure 10a shows the absolute wavelength shift produced by the tested diluted solutions for several etching times within the range from 289 min to 302 min as a function of the dilution. A highly linear response is obtained indicating a sensitivity ranging from −0.19214 nm/% to −0.42871 nm/% for etching times ranging from 289 min to 302 min. The largest wavelength shift, which was obtained for the largest etching time (302 min), was −20.5 nm, as can be confirmed from Figure 10a. The sensor sensitivity as a function of the thickness of the cladding around the off-axis core is also shown in Figure 10b. In this particular case, since the reference spectral signal (pure tequila) blue shifts as the tequila concentration decreases, a more negative sensitivity is obtained as the cladding becomes thinner. These results basically confirm the behavior exhibited in the previous cases from the point of view that a larger spectral shift is achieved for larger etching times but, unlike the previous cases, the negative sign makes reference to the spectral shift towards smaller wavelengths.

We should mention that the sensor is capable of measuring higher RI up to a value of 1.445. In fact, as shown in Figure 3b, as the RI of the liquid approaches the RI of the cladding the coupling coefficient changes quite rapidly and hence the sensitivity should also increase significantly. In the case of RI values higher than 1.445, even when the sensitivity is enhanced, the losses will be also significant and the contrast of the spectral response is dramatically reduced making very difficult to follow the shift of either a peak or valley.

Temperature compensation is an issue that needs to be considered just as with any other fiber based sensor. When dealing with RI sensor this is a critical issue since all the sets of liquids used in this work, the binary liquid mixtures between water and ethylene glycol, the saline solutions for both high- and low-concentration regime, and the diluted solutions of tequila, are dependent on temperature this effect needs to be taken into account in order to correct the spectral shift of the TCF response. In order to estimate the temperature dependence of the TCF, temperature was measured in the range from 23 °C to 100 °C, resulting in a total spectral red shift of approximately 3.3 nm, which is to be expected due to the positive thermo-optic coefficient of fused silica. The linear fit of the experimental measurements suggest a temperature sensitivity of 42.88 ± 1.18 pm/°C, which is good agreement with the sensitivity reported for a high-temperature sensor based on this same TCF [23]. Based on the fact that most of the experimental measurements performed result in spectral shifts on the order of several nm, which is approximately three orders of magnitude larger than that obtained due to temperature, temperature effects can be neglected in such cases. In the case of smaller spectral shifts, and given the linearity of the temperature dependence of the sensor, thermal effects can be easily eliminated by knowing the temperature.

## 4. Conclusions

In summary, a novel and simple fiber optic sensor for high sensitivity RI measurements based on a TCF was demonstrated. The TCF is forced to interact with the external environment by controllably removing the cladding around the off-axis core of the TCF. This allows not only inducing the interaction between the TCF and the surrounding media but also adjusting the sensitivity of the TCF-based RI sensor by only controlling the duration of the etching process. The proposed experimental setup allows neglecting bending and surface tension effects which makes the sensor suitable for operating at different RI ranges and for a wide variety of liquids. In this regard, the high performance of the proposed RI sensor was demonstrated and two potential applications, namely the measurement of salinity in both high- and low-concentration regimes, and the detection of adulterated alcoholic beverages were investigated.

The sensor spectral response was measured using the sets of liquids after several etching times and, in order to disclose how the sensor sensitivity changes within the RI range of interest, the sensitivity was characterized by measuring the absolute spectral shift induced by the surrounding liquids. The largest sensitivity experimentally demonstrated was 3,119 nm/RIU for the RI range from 1.3160 to 1.3943. However, from the numerical fitting of the experimental results, it was found that the sensitivity can be enhanced up to an approximate value of 3485.67 nm/RIU for the same refractive index range. Compared to RI sensors based on non-interferometric techniques, FBG and LPFG, and the use of PCF, the proposed sensor exhibits a higher sensitivity which is around 1.5 times larger [5–7]. The sensor has been demonstrated to be simple, robust, and having an adjustable sensitivity, which makes the sensor suitable to operate over a wide RI range and still perform RI measurements with high sensitivity without requiring any special equipment to fabricate the sensor. Furthermore, the length of the TCF section can be changed in order to maximize the free-spectral range according to the RI that will be measured.
